# The 150 most important questions in cancer research and clinical oncology series: questions 86–93: Edited by *Chinese Journal of Cancer*

**DOI:** 10.1186/s40880-018-0266-3

**Published:** 2018-01-22

**Authors:** Chinese Journal of Cancer

**Affiliations:** Sun Yat-sen University Cancer Center, Guangzhou, 510060 Guangdong P. R. China

**Keywords:** Supportive care, Animal model, Mimic immunotherapy, Hepatitis B virus-associated cancer, Non-hepatocellular cancer, Tumor metabolism, Prophylactic therapy, Postoperative radiotherapy, Survival, Molecular event, Oral cancer, Tumor genome

## Abstract

Since the beginning of 2017, *Chinese Journal of Cancer* has published a series of important questions in cancer research and clinical oncology, which spark diverse thoughts, interesting communications, and potential collaborations among researchers all over the world. In this article, 8 more questions are presented as follows. Question 86. In which circumstances is good supportive care associated with a survival advantage in patients with cancer? Question 87. Can we develop animal models to mimic immunotherapy response of cancer patients? Question 88. What are the mechanisms underlying hepatitis B virus-associated non-hepatocellular cancers? Question 89. Can we more precisely target tumor metabolism by identifying individual patients who would benefit from the treatment? Question 90. What type of cranial irradiation-based prophylactic therapy combination can dramatically improve the survival of patients with extensive small-cell lung cancer? Question 91. How can postoperative radiotherapy prolong overall survival of the patients with resected pIIIA-N2 non-small cell lung cancer? Question 92. What are the key molecular events that drive oral leukoplakia or erythroplakia into oral cancer? Question 93. How could we track the chemotherapeutics-driven evolution of tumor genome in non-small cell lung cancer for more effective treatment?

## Text

To accelerate our endeavors to overcome cancer, *Chinese Journal of Cancer* has launched a program of publishing 150 most important questions in cancer research and clinical oncology [1]. Since the beginning of 2017, *Chinese Journal of Cancer* has published a series of important questions in cancer research and clinical oncology [2–12], which spark diverse thoughts, interesting communications, and potential collaborations among researchers all over the world. In this article, Questions 86–93 are selected and presented. This program of collecting and publishing the key questions is still ongoing. Please send your thoughtful questions to Ms. Ji Ruan via email: ruanji@sysucc.org.cn.

## Question 86: In which circumstances is good supportive care associated with a survival advantage in patients with cancer?

### Background and implications

It is well documented that good supportive care throughout the treatment and survival phases of cancer as well as palliative care towards the end of life improve the quality of life of the patients [13]. In some circumstances, good supportive care may also prolong survival. Quintin et al. [14] performed a global analysis of data from multiple trials and showed that quality of life and presenting symptoms were prognostic factors for survival of patients with cancer in addition to other clinical characteristics. For example, febrile neutropenia following chemotherapy is a life-threatening adverse effect and can be mitigated by giving the chemotherapy with granulocyte colony stimulating factor (G-CSF). It is well documented that mortality from infection is reduced by G-CSF [15]; however, it is not clear that this may be translated into an overall survival advantage. Prophylactic use of antiemetics increases the tolerance of chemotherapy, allowing full dose to be given and courses of chemotherapy to be completed, which has been shown to prolong survival [16]. Good symptom control with chemotherapy may also prolong survival. In a randomized study, second-line chemotherapy was given with or without early palliative care to patients with non-small cell lung cancer, and the results showed that those receiving the palliative care in addition to their chemotherapy had significantly longer survival than those receiving chemotherapy only (11.6 vs. 8.9 months, *P* = 0.02) [17]. Further, it is intriguing that psychosocial support may prolong survival. A weekly psychosocial support group and self-hypnosis for pain was added to anticancer therapy for breast cancer patients in a randomized trial and resulted in prolonged survival as compared with those who only received anticancer therapy [18]. The relationship between social networks and social support has been equivocal although a large breast cancer study showed an increase in both all-cause mortality and breast cancer mortality in women who are socially isolated [19, 20]. Certainly, the narratives of exceptional survivors of incurable cancer ascribed some of their outcomes to family support [21].

Clearly, in some circumstances, the addition of good supportive care which addresses cancer-associated symptoms and adverse effects of treatment can be added to anticancer treatment to prolong survival. More researches are needed to better define when this occurs.

#### Submitter

Ian Olver.

#### Affiliation and email

Sansom Institute for Health Research, University of South Australia, Adelaide, South Australia 5001, Australia.

ian.olver@unisa.edu.au.

## Question 87: Can we develop animal models to mimic immunotherapy response of cancer patients?

### Background and implications

Efforts on immuno-oncology (I/O) research to fight cancer are in exponential phase of growth due to recent breakthrough in the development of immune checkpoint inhibitors and unprecedented rate of regulatory approval to shorten the otherwise lengthy bench to bedside process. The prevalent models include syngeneic, genetically engineered, and partially humanized mouse models each with its advantages and limitations. The lack of precise animal models that would be capable of mimicking human immune microenvironment is one of the major challenges for proper preclinical evaluation of I/O therapies and identifying patients most likely to be benefited from specific I/O strategies.

The ideal animal models should also possess effective biomarkers for monitoring the immune functions of the host as well as therapeutic effects of I/O. In current clinical practice, the remarkable progress in the development of immune checkpoint inhibitors went solo without parallel advancement of definitive patient selection tool. The cost, toxicities, and the time delay for the 40%–60% of patients not benefiting from immunotherapy makes it imperative to identify valid prognostic biomarkers [e.g., programmed death-ligand 1 (PD-L1) expression, mismatch repair (MMR) deficiency, cluster of differentiation 8 (CD8) T cell infiltrates, tumor mutation burden] that could predict patient response and facilitate differentiation of durable response versus transient response. Given the dynamic nature of the immune response and the complexity of immune/tumor interaction, development of biomarkers for immunotherapies is highly challenging. Presence of tumor-specific antigens, expression of immunosuppressive molecules [PD-L1, indoleamine 2,3-dioxygenase (IDO), and so on] by tumor cells, and mutation load and landscape all contribute to the response of tumor cells to I/O therapies. While most of the biomarker-searching efforts had focused on tumor characteristics, the role of host immune system is equally important. The effectiveness of a given immunotherapeutic approach depends on a pre-existing immune state of a patient.

In summary, development of clinically relevant animal models possessing discerning prognostic markers is critical to fulfill the promise of immunotherapy as a paradigm-shifting strategy to fight the most aggressive and intractable cancers.

#### Submitters

Qian Shi and Meng Qiao.

#### Affiliation and emails

Crown Biosciences Inc., Taicang, Jiangsu Province, 215400, P. R. China.

shiqian@crownbio.com; qiaomeng@crownbio.com.

## Question 88: What are the mechanisms underlying hepatitis B virus-associated non-hepatocellular cancers?

### Background and implications

Hepatitis B virus (HBV) infection is a strong risk factor for the development of hepatocellular carcinoma. Epidemiological studies have also shown that HBV infection may increase the incidence of several types of non-hepatocellular cancers, including gastric adenocarcinoma, pancreatic ductal carcinoma, and non-Hodgkin lymphoma (NHL). Clinical studies further suggested that some of these HBV-associated non-hepatocellular cancers, for instance a subtype of NHL, diffuse large B cell lymphoma, exhibit a more aggressive disease course with poor prognosis, independent of its pathological subtype. However, what are the mechanisms underlying these associations and whether the viral infection is indeed disease-causing or rather a contributing co-factor remain unclear. Two major hypotheses, direct viral infection of the corresponding cell types and chronic viral antigen stimulations, have been proposed. In both scenarios, infection may result in dysregulation of host cellular processes and increased genome instability, and in the case of direct infection, like in hepatocellular carcinoma, integration of viral DNA into the host genome may lead to activation of selective oncogenes. More detailed morphological and molecular studies, including characterization of the genome of these HBV-associated non-hepatocellular cancers and the repertories of infiltrating immune cells, may provide further clues to this question. It will also be of interest to determine if there is an association between genotype (strain of HBV) and phenotype (type of cancer). Finally, in areas/countries with a high prevalence of infection and initiated the mandatory HBV vaccine program decades ago, theoretically, the incidence of these non-hepatocellular cancers should decrease with time. Of note, this may be complicated by the increased contribution of other risk factors, especially life style-related factors. Chronic HBV infection is endemic in some parts of Asia, Africa, and South America and remains to be a public health burden in these areas. Further understanding the molecular mechanisms underlying the HBV-associated cancers will help us to develop novel or more precise therapies for the affected patients.

#### Submitters

Yao Liu and Qiang Pan-Hammarström.

#### Affiliation and emails

Division of Clinical Immunology, Department of Laboratory Medicine, Karolinska University Hospital, Huddinge, Stockholm SE 141 86, Sweden.

yao.liu@ki.se; Qiang.Pan-Hammarstrom@ki.se.

## Question 89: Can we more precisely target tumor metabolism by identifying individual patients who would benefit from the treatment?

### Background and implication

During the process of tumorigenesis, tumor cells must face two challenges: first, obtaining the nutrients needed for the rapid growth; and second, evading the surveillance and attack from the host immune system. Tumor cell’s unique metabolic program can be used to meet these challenges. Glycolysis is the major metabolic process used by malignant tumors, even when oxygen supply is adequate, which is termed as “the Warburg effect”. Glycolysis decreases the pH value of the tumor microenvironment (TME); therefore, tumor cells can inhibit the activities of antigen-presenting cells (APCs) and cytotoxic T lymphocytes (CTLs) by controlling the acidity of TME, eventually leading to tumor cell immune escape. A second group of metabolism-related modification directly targets the major histocompatibility complex-I (MHC-I) and related molecules and hence sensitizes cancer cells to the cytolytic actions of the anti-tumor adaptive immune response.

Recent findings from in vitro and in vivo studies have shown that targeting tumor and immune cell metabolism hold the promising possibilities toward clinical therapeutics for treating cancer [22, 23]. However, clinical benefit has only been observed in a small number of patients [24–28]. Most patients still do not respond to these new therapies, and nearly all patients with certain types of cancer (i.e., pancreatic and colorectal cancers) do not respond. The reason is probably because tumor metabolism may vary over the course of tumor development, or some hidden tumor metabolic products modulate signaling pathways important for immune cell activation. A new hypothesis has been proposed that tumor cells can change their metabolism by waves of gene regulation to adjust to their different needs [29]. Some of these waves are originated by deregulated expression of oncogenes, which have already been linked to metabolic remodeling. On the other hand, different parts of solid tumor sometimes possess different epigenetic characteristics and may be derived from distinct cancer stem cell populations. Therefore, the most serious challenge in reshaping the tumor-specific metabolism and immune profiles in TME is to understand the metabolic heterogeneity which is extremely complicated depending not only on tumor and immune cell types but also on tumor stages and subset of patient population.

Nevertheless, the success associated with these new approaches has opened new investigations addressing several questions: How much metabolism pathways represent true vulnerabilities for tumor development and immunosuppression in different types and stages of cancer? Are there other factors that may be blocking, even temporarily, which is critical for tumor control? How different subsets of tumor cell populations respond to metabolic intervention? Can we identify ahead of time the patients who would benefit from metabolic targeted therapy?

Notably, tumor and immune cells share similar metabolic needs and reprogramming during proliferation to support their increased biosynthetic and energy demands [30, 31] and often compete for the same nutrients. Therefore, deprivation of nutrients in TME must be cautiously explored to eliminate potential negative impacts on the anti-tumor immunity. Understanding the underlying mechanisms of metabolic interplay between tumor and immune cells will provide new precise directions to manipulate the tumor metabolism for better treatment outcome.

#### Submitters

Jianyang Wang and Zhouguang Hui.

#### Affiliation and emails

Department of Radiation Oncology (JW and ZH), Department of VIP Medical Services (ZH), National Cancer Center/Cancer Hospital, Chinese Academy of Medical Sciences & Peking Union Medical College, Beijing 100021, China.

pkucell@163.com; drhuizg@163.com.

## Question 90: What type of cranial irradiation-based prophylactic therapy combination can dramatically improve the survival of patients with extensive small-cell lung cancer?

### Background and implication

Brain metastasis is a common reason of treatment failure in small-cell lung cancer (SCLC), particularly in extensive disease which represents approximately two-thirds of newly diagnosed SCLCs. Recent studies have found that thoracic radiotherapy (TRT) can increase the 2-year overall survival (OS) rate of patients with extensive SCLC after chemotherapy [32–34]. However, it remains controversial that whether prophylactic cranial irradiation (PCI) can prolong OS [35–38]. The combination of TRT and PCI may boost the chances of survival, but there will be not much predictable OS benefit even if more prospective studies with large sample sizes are conducted. After first-line chemotherapy, the comprehensive treatment based on TRT and PCI, such as combining with new anti-metastatic drugs, will make great strides toward OS improvement. The application of more accurately targeted therapy is now available and promising. Maintenance treatment with sunitinib can prolong progression-free survival (PFS) in extensive SCLC [39]. Recent studies on new drugs targeting the signaling pathways (e.g., Notch signaling) related to neuroendocrine differentiation, DNA reparation, and immune checkpoint are ongoing. The Notch signaling pathway influences multiple processes in normal cell morphogenesis, including the differentiation of multipotent progenitor cells (neuron differentiation), cell apoptosis, and cell proliferation. Rovalpituzumab tesirine (Rova-T) targeting the Notch signaling pathway showed promising results in a phase I trial [40]. Poly ADP-ribose polymerase (PARP) is DNA repairase and is critical in DNA damage repair. By inhibiting PARP, proliferation of malignant cells can be suppressed. Veliparib, a PARP inhibitor, has yielded antitumor activity in SCLC [41]. Researches on immunotherapy primarily focus on cytotoxic T-lymphocyte-associated antigen-4 (CTLA-4) and programmed cell death protein 1 (PD-1)/programmed death ligand 1 (PD-L1) inhibitors. Nivolumab alone and in combination with ipilimumab resulted in encouraging response rates (RR) in a phase I/II trial in the relapsed tumor setting [42]. The development of anti-metastasis agents is clearly critical for further improving the survival benefits of the patients with extensive SCLC. In addition, advanced irradiation technique is expected to be adopted in future clinical trails to decrease irradiation-induced injury in hippocampus for protecting cognitive function [43].

#### Submitters

Lei Deng and Zhouguang Hui.

#### Affiliation and emails

Department of Radiation Oncology (LD and ZH), Department of VIP Medical Services (ZH), National Cancer Center/Cancer Hospital, Chinese Academy of Medical Sciences & Peking Union Medical College, Beijing 100021, China.

dengleipumc@163.com; drhuizg@163.com.

## Question 91: How can postoperative radiotherapy prolong overall survival of the patients with resected pIIIA-N2 non-small cell lung cancer?

### Background and implication

For patients with resected pIIIA-N2 non-small cell lung cancer (NSCLC), the main reason of treatment failure is locoregional and/or distant relapse. Adjuvant chemotherapy can prolong overall survival to some extent. However, the role of postoperative radiotherapy is not well defined.

A meta-analysis study on postoperative radiotherapy published in 1998 concluded that postoperative radiotherapy did not prolong the survival, even in patients with stage III and pN2 NSCLC, which may due to the toxicities with suboptimal, outdated irradiation equipment and techniques [44]. Improvements in conformal radiotherapy techniques have led to a resurgence of interest in studying the effect of postoperative radiotherapy on pIIIA-N2 NSCLC. Several retrospective, large-size, case–control studies have shown that postoperative radiotherapy using three dimensional conformal radiotherapy (3D-CRT) or intensity modulated radiation therapy (IMRT) techniques can prolong overall survival [45]. However, the benefit still needs to be confirmed by randomized clinical trials (RCTs). Up to now, there are three such phase III RCTs. CALGB 9734, the earliest one, failed because of slow accrual [46]. LUNGART, the ongoing one, began in 2007 and aims to enroll 700 patients by its conclusion in 2022. The other ongoing phase III multi-center RCT (NCT00880971), conducted by our institute, has accrued 400 patients over planned 500 patients. However, due to the heterogeneity of pIIIA-N2 NSCLC, only certain subgroups of patients may benefit from postoperative radiotherapy. Selecting suitable candidates or the populations at high risk who may benefit from postoperative radiotherapy is the next and profound task.

It is expected that by combining with targeted therapy and/or immunotherapy, the therapeutic effects of postoperative radiotherapy can be enhanced. For patients with completely resected NSCLC with epidermal growth factor receptor (EGFR) activating mutation, two recently reported RCTs have showed that adjuvant EGFR tyrosine kinase inhibitors (TKIs) significantly prolonged disease-free survival as compared with adjuvant chemotherapy [47, 48]. Therefore, for pIIIA-N2 NSCLC patients with EGFR-activating mutation receiving EGFR TKIs, the value of postoperative radiotherapy should be further evaluated. Theoretically, any new agent that can inhibit metastasis could enhance the efficacy of postoperative radiotherapy, and more efforts are warranted in this direction.

#### Submitters

Yu Men and Zhouguang Hui.

#### Affiliation and emails

Department of VIP Medical Services, National Cancer Center/Cancer Hospital, Chinese Academy of Medical Sciences & Peking Union Medical College, Beijing 100021, China.

sharon0303@126.com; drhuizg@163.com.

## Question 92: What are the key molecular events that drive oral leukoplakia or erythroplakia into oral cancer?

### Background and implications

The natural history of cancer is poorly understood. The main reason is that in the vast majority of the cases, malignant tumors are diagnosed after becoming clinically perceptible. The paradox is that, for patients dying from cancer, the time from diagnosis to death is often much shorter than the long period preceding diagnosis. Most of our knowledge is based on the analysis of established malignant tumors in comparison with histologically normal tissue, and the use of naturally occurring or genetically engineered animal models that may not recapitulate the natural history of human cancer. Initiation is thought to be the first step of the multistep model of cancer development, followed by promotion and progression. However, the stepwise and sequential progression model is being challenged by some clinical observations. One of the best examples is the natural history of oral leukoplakia or erythroplakia, the most frequent, potentially malignant lesions of the oral cavity. They can remain for many years without changing, can regress spontaneously or after cessation of tobacco smoking, alcohol drinking, or smokeless tobacco, and can transform to invasive squamous cell carcinoma (SCC) at the same site or at distance from the potentially malignant lesion. The reported rate of malignant transformation has been low in community-based studies in developing countries (0.06% per year) and higher in observational studies in western countries involving patients followed in hospital-based academic centers (1%–5% per year) [49].

We believe that the longitudinal and spatial dynamics of early-stage tumorigenesis in the oral cavity through comprehensive evaluation of cellular and molecular changes in the epithelial and stromal cells represent a unique setting to get more insight into the natural history of carcinomas. The disease is prevalent in different parts of the world and associated with various environmental agents: in western countries, it frequently affects patients with smoking and alcohol drinking history in the form of oral leukoplakia, whereas in Southeast Asia it frequently affects patients consuming areca nut, betel leaf, and quid who preferentially develop erythroplakia. Of note, oral potentially malignant lesions and SCC negative for human papillomavirus affecting patients with no smoking or alcohol drinking history, although representing a minority of all patients, have an increasing incidence over the past decades for unknown reasons. The oral cavity is easily accessible, and it is considered to be a molecular mirror of molecular alterations induced by smoking in the upper and lower aerodigestive tract. Prospectively validated in situ biomarkers of risk (e.g., loss of heterozygocity at prespecified chromosomal sites) can be used to define cohorts of patients with potentially malignant lesions at high risk of developing oral cancer. These elements represent a strong rationale for intensive exploration in this unique setting. It has the potential to foster international collaborations toward the better understanding of the biology of early-stage tumorigenesis, and provide an opportunity to develop personalized prevention strategies that will benefit patients far beyond the decreased incidence of oral cancer.

#### Submitter

Pierre Saintigny.

#### Affiliation and email

Univ Lyon, Université Claude Bernard Lyon 1, INSERM 1052, CNRS 5286, Centre Léon Bérard, Centre de recherche en cancérologie de Lyon; Department of Translational Research and Innovation, and Department of Medical Oncology, Centre Léon Bérard, Lyon, 69008, France.

Pierre.SAINTIGNY@lyon.unicancer.fr.

## Question 93: How could we track the chemotherapeutics-driven evolution of tumor genome in non-small cell lung cancer for more effective treatment?

### Background and implication

Currently, effective drug treatments for the patients with non-small cell lung cancer (NSCLC) comprise mainly of standard platinum-based cytotoxic treatment, targeted therapies including inhibitors for epidermal growth factor receptor (EGFR) and anaplastic lymphoma kinase (ALK), and immunotherapy. However, treatment resistance will inevitably occur in most patients after a certain period of time. This is believed to be partially caused by the heterogeneity in tumor genome. Cancer is a genomic disease, and cancer genome constantly undergoes changes under selective pressure from anticancer drug treatment. This alteration is also named tumor evolution, which partially explains acquired drug resistance. For example, some acquired secondary mutations, e.g., EGFR C797S, have been detected in the patient who initially harbors EGFR T790M mutation when resistance against first-line EGFR inhibitor occurs. Therefore, there is a need to dynamically monitor tumor clonal evolution in NSCLC patients. Methods for monitoring tumor evolution include multiregional sequencing and liquid biopsies. In multiregional sequencing, tumor masses from several regions are sequenced in parallel through next-generation sequencing. In liquid biopsies, a serial of circulating molecules or cells in the blood including circulating tumor DNAs (ctDNAs) and circulating tumor cells (CTCs) could reflect the information of tumor genome. These methods could represent the whole tumor genomic landscape and reflect tumor heterogeneity. In addition to this, longitudinal or serial monitoring tumor genome through liquid biopsies or multiregional sequencing could keep track of the tumor genome in both time and space. Of course, it remains a technical challenge in collecting biopsy samples from multiple time points in the same patient. Advances in imaging-guided transthoracic biopsy of lung lesions are the hope for delivering personalized treatments in response to the evolving tumor genome for dramatically improving treatment outcomes in NSCLC patients.

#### Submitters

Yi Xiong and Zhi-Xing Lu.

#### Affiliation and emails

Xiangya school of medicine, Central South University, Changsha, Hunan 410008, P. R. China.

xiongyi123@csu.edu.cn; Luzhixing@csu.edu.cn.

## References

[1] Qian CN, Zhang W, Xu RH (2016). Defeating cancer: the 150 most important questions in cancer research and clinical oncology. Chin J Cancer..

[2] Wee JT, Poh SS (2017). The most important questions in cancer research and clinical oncology. Question 1. Could the vertical transmission of human papilloma virus (HPV) infection account for the cause, characteristics, and epidemiology of HPV-positive oropharyngeal carcinoma, non-smoking East Asian female lung adenocarcinoma, and/or East Asian triple-negative breast carcinoma?. Chin J Cancer..

[3] Venniyoor A (2017). The most important questions in cancer research and clinical oncology: question 2–5. Obesity-related cancers: more questions than answers. Chin J Cancer..

[4] Chinese Journal of Cancer (2017). The 150 most important questions in cancer research and clinical oncology series: questions 6–14. Chin J Cancer..

[5] Chinese Journal of Cancer (2017). The 150 most important questions in cancer research and clinical oncology series: questions 15–24. Chin J Cancer..

[6] Chinese Journal of Cancer (2017). The 150 most important questions in cancer research and clinical oncology series: questions 25–30. Chin J Cancer..

[7] Chinese Journal of Cancer (2017). The 150 most important questions in cancer research and clinical oncology series: questions 31–39. Chin J Cancer..

[8] Chinese Journal of Cancer (2017). The 150 most important questions in cancer research and clinical oncology series: questions 40–49. Chin J Cancer..

[9] Chinese Journal of Cancer (2017). The 150 most important questions in cancer research and clinical oncology series: questions 50–56. Chin J Cancer..

[10] Chinese Journal of Cancer (2017). The 150 most important questions in cancer research and clinical oncology series: questions 57–66. Chin J Cancer..

[11] Chinese Journal of Cancer (2017). The 150 most important questions in cancer research and clinical oncology series: questions 67–75. Chin J Cancer..

[12] Chinese Journal of Cancer (2017). The 150 most important questions in cancer research and clinical oncology series: questions 76–85. Chin J Cancer..

[13] Rosenbaum E, Gautier H, Fobair P, Neri E, Festa B, Hawn M, Andrews A, Hirschberger N, Selim S, Spiegal D (2004). Cancer supportive care, improving the quality of life for cancer patients. A program evaluation report. Support Care Cancer.

[14] Quintin C, Martinelli F, Coeans C (2014). A global analysis of multitrial data investigating quality of life and symptoms as prognostic factors for survival in different tumour sites. Cancer.

[15] Kuderer NM, Dale DC, Crawford J, Lyman GH (2007). Impact of primary prophylaxis with granulocyte colony-stimulating factor on febrile neutropenia and mortality in adult cancer patients receiving chemotherapy: a systematic review. J Clin Oncol.

[16] Chan VTC, Yeo W (2011). Antiemetic therapy options for chemotherapy-induced nausea and vomiting in breast cancer patients. Breast Cancer Targets Ther.

[17] Temel JS, Greer JA (2010). Early palliative care for patients with metastatic non-small cell lung cancer. New Engl J Med.

[18] Spiegel D, Bloom JR, Kraemer HC, Gottheil E (1989). Effect of psychosocial treatment on survival of patients with metastatic breast cancer. Lancet.

[19] Eli K, Nishimoto R, Mediansky L, Mantell J, Hamovitch M (1992). Social relations, social support and survival among patients with cancer. J Psychosom Res.

[20] Kroenke CH, Kubznsky LD, Schernhammer ES, Holmes LD, Kawachi I (2008). Social networks, social support, and survival after breast cancer diagnosis. J Clin Oncol.

[21] Frenkel M, Gross S, Popper Giveon A, Sapire K, Hermoni D (2015). Living outliers: experiences, insights and narratives of exceptional survivors of incurable cancer. Future Oncol.

[22] Luciani F (2004). Effect of proton pump inhibitor pretreatment on resistance of solid tumors to cytotoxic drugs. J Natl Cancer Inst.

[23] Zhang Y (2014). Involvement of metformin and AMPK in the radioresponse and prognosis of luminal versus basal-like breast cancer treated with radiotherapy. Oncotarget.

[24] Boroughs LK, DeBerardinis RJ (2015). Metabolic pathways promoting cancer cell survival and growth. Nat Cell Biol.

[25] Biswas SK (2015). Metabolic reprogramming of immune cells in cancer progression. Immunity.

[26] Xing Y (2015). Metabolic reprogramming of the tumour microenvironment. FEBS J.

[27] Wang BY (2015). Intermittent high dose proton pump inhibitor enhances the antitumor effects of chemotherapy in metastatic breast cancer. J Exp Clin Cancer Res.

[28] Psutka SP (2015). The association between metformin use and oncologic outcomes among surgically treated diabetic patients with localized renal cell carcinoma. Urol Oncol.

[29] Smolkova K (2011). Waves of gene regulation suppress and then restore oxidative phosphorylation in cancer cells. Int J Biochem Cell Biol.

[30] Macintyre AN, Rathmell JC (2013). Activated lymphocytes as a metabolic model for carcinogenesis. Cancer Metab.

[31] Marelli-Berg FM, Fu H, Mauro C (2012). Molecular mechanisms of metabolic reprogramming in proliferating cells: implications for T-cell-mediated immunity. Immunology.

[32] Jeremic B, Shibamoto Y, Nikolic N (1999). Role of radiation therapy in the combined-modality treatment of patients with extensive disease small-cell lung cancer: a randomized study. J Clin Oncol.

[33] Zhu H, Zhou Z, Wang Y (2011). Thoracic radiation therapy improves the overall survival of patients with extensive-stage small cell lung cancer with distant metastasis. Cancer.

[34] Slotman BJ, van Tinteren H, Praag JO (2015). Use of thoracic radiotherapy for extensive stage small-cell lung cancer: a phase 3 randomised controlled trial. Lancet.

[35] Auperin A, Arriagada R, Pignon JP (1999). Prophylactic cranial irradiation for patients with small-cell lung cancer in complete remission. Prophylactic cranial irradiation overview collaborative Group. N Engl J Med.

[36] Meert AP, Paesmans M, Berghmans T (2001). Prophylactic cranial irradiation in small cell lung cancer: a systematic review of the literature with meta-analysis. BMC Cancer.

[37] Slotman B, Faivre-Finn C, Kramer G (2007). Prophylactic cranial irradiation in extensive small-cell lung cancer. N Engl J Med.

[38] Takahashi T, Yamanaka T, Seto T (2017). Prophylactic cranial irradiation versus observation in patients with extensive-disease small-cell lung cancer: a multicentre, randomised, open-label, phase 3 trial. Lancet Oncol..

[39] Ready NE, PangHH GuL (2015). Chemotherapy with or without maintenance sunitinib for untreated extensive-stage small-cell lung cancer: a randomized, double-blind, placebo-controlled phase II study-CALGB 30504 (alliance). J Clin Oncol..

[40] Rudin CM, Pietanza MC, Bauer TM (2017). Rovalpituzumab tesirine, a DLL3-targeted antibody-drug conjugate, in recurrent small-cell lung cancer: a first-in-human, first-in-class, open-label, phase 1 study. Lancet Oncol..

[41] Byers LA, Krug LM, Waqar SN (2017). Improved small cell lung cancer (SCLC) response rates with veliparib and temozolomide: results from a Phase II trial. J Thorac Oncol..

[42] Antonia SJ, López-Martin JA, Bendell J (2016). Nivolumab alone and nivolumab plus ipilimumab in recurrent small-cell lung cancer (CheckMate 032): a multicentre, open-label, phase 1/2 trial. Lancet Oncol..

[43] Gondi V, Hermann BP, Mehta MP (2013). Hippocampal dosimetry predicts neurocognitive function impairment after fractionated stereotactic radiotherapy for benign or low-grade adult brain tumors. Int J Radiat Oncol Biol Phys.

[44] PORT Meta-analysis Trialists Group (1998). Postoperative radiotherapy in non-small-cell lung cancer: systematic review and meta-analysis of individual patient data from nine randomised controlled trials. Lancet (London, England).

[45] Patel SH, Ma Y, Wernicke AG, Nori D, Chao KS, Parashar B (2014). Evidence supporting contemporary post-operative radiation therapy (PORT) using linear accelerators in N2 lung cancer. Lung Cancer (Amsterdam, Netherlands).

[46] Perry MC, Kohman LJ, Bonner JA, Gu L, Wang X, Vokes EE (2007). A phase III study of surgical resection and paclitaxel/carboplatin chemotherapy with or without adjuvant radiation therapy for resected stage III non-small-cell lung cancer: cancer and leukemia group B 9734. Clin Lung Cancer.

[47] Wu Y, Zhong W, Wang Q, Xu S, Mao W, Wu L (2017). Gefitinib (G) versus vinorelbine + cisplatin (VP) as adjuvant treatment in stage II–IIIA (N1–N2) non-small-cell lung cancer (NSCLC) with EGFR-activating mutation (ADJUVANT): a randomized, phase III trial (CTONG 1104). J Clin Oncol.

[48] Yue D, Xu S, Wang Q, Li X, Shen Y, Zhao H, et al. Efficacy and safety of erlotinib vs vinorelbine/cisplatin as adjuvant therapy for stage IIIA EGFR mutant NSCLC patients. In: IASLC 18th world conference on lung cancer. 2017. p. 134.

[49] Patel SC, Carpenter WR, Tyree S, Couch ME, Weissler M, Hackman T (2011). Increasing incidence of oral tongue squamous cell carcinoma in young white women, age 18 to 44 years. J Clin Oncol.

